# A mathematical model of cartilage regeneration after chondrocyte and stem cell implantation – II: the effects of co-implantation

**DOI:** 10.1177/2041731419827792

**Published:** 2019-03-15

**Authors:** Kelly Campbell, Shailesh Naire, Jan Herman Kuiper

**Affiliations:** 1 School of Computing and Mathematics, Keele University, Keele, UK; 2 Institute for Science and Technology in Medicine, Keele University, Keele, UK; 3 Robert Jones and Agnes Hunt Orthopaedic & District Hospital NHS Trust, Oswestry, UK

**Keywords:** Mathematical modelling, cartilage defect, regenerative medicine, co-culture, mesenchymal stem cells

## Abstract

We present a mathematical model of cartilage regeneration after cell therapy, to show how co-implantation of stem cells (mesenchymal stem cells) and chondrocytes into a cartilage defect can impact chondral healing. The key mechanisms involved in the regeneration process are simulated by modelling cell proliferation, migration and differentiation, nutrient diffusion and Extracellular Matrix (ECM) synthesis at the defect site, both spatially and temporally. In addition, we model the interaction between mesenchymal stem cells and chondrocytes by including growth factors. In Part I of this work, we have shown that matrix formation was enhanced at early times when mesenchymal stem cell-to-chondrocyte interactions due to the effects of growth factors were considered. In this article, we show that the additional effect of co-implanting mesenchymal stem cells and chondrocytes further enhances matrix production within the first year in comparison to implanting only chondrocytes or only mesenchymal stem cells. This could potentially reduce healing time allowing the patient to become mobile sooner after surgery.

## Introduction

Autologous chondrocyte implantation (ACI) is the most commonly used cell-based therapy to treat chondral defects.^1^ The treatment comprises obtaining chondrocytes from a small harvest of healthy cartilage, a period of culturing the chondrocytes to expand their numbers, and implantation of these cells into the defect under a membrane. It does have some drawbacks, in particular the need for an extra knee surgery procedure to harvest the chondrocytes, difficulties in obtaining an adequate number of chondrocytes and donor site morbidity.^2^ Mesenchymal stem cells (MSCs) are increasing in popularity as a cell source for regenerative medicine approaches for cartilage regeneration, such as cell implantation to treat articular cartilage defects of the joints.^3^ The benefits of using MSCs instead of chondrocytes have been well documented, including their larger availability within the body and their ability to undergo chondrogenesis and deposit matrix under the influence of growth factors.^4^

An alternative cell-based therapy, denoted here as articular stem cell implantation (ASI), reproduces the approach of culture expansion and implantation, except MSCs are used instead of chondrocytes.^2^ Lutianov et al.^5^ developed a mathematical model to simulate and compare the repair of a chondral defect with new cartilage following implantation of either chondrocytes (ACI) or MSCs (ASI) along the bottom of the defect. This model assumed that following implantation, MSCs only contributed to cartilage formation via their differentiation into chondrocytes. One difference between ACI and ASI according to this model was that cartilage formation after ASI started later than after ACI. As is now widely recognised, MSCs used in this way do not only contribute to the repair process via their differentiation into chondrocytes but also via their secretion of growth factors and cytokines, termed as their ‘trophic’ effect.^6,7^ Work by Wu^8^ identifies two growth factors, Fibroblast Growth Factor 1 (FGF-1) and Bone Morphogenetic Protein 2 (BMP-2), as particularly important during cartilage regeneration. These two growth factors were identified when investigating the effect of co-cultures of MSCs and chondrocytes on cartilage formation.^8^ They are released by MSCs and chondrocytes and mediate MSC-to-chondrocyte interaction, enhancing chondrocyte proliferation and MSC chondrogenesis (see Figure 3 for a schematic of this cell-to-cell interaction in Part I of our work^9^). Their observations were modelled mathematically in Part I, which studied the effects of these growth factors after MSC implantation (ASI) into the defect.^9^ Our simulations showed that matrix formation following ASI was enhanced at early times when cell-to-cell interactions mediated by these growth factors were taken into account. This was mainly due to the presence of BMP-2, resulting in increased formation of chondrocytes via increased chondrocyte proliferation and MSC chondrogenesis, and hence enhancing early matrix production in comparison to the case when no growth factors are present. At later time points, no differences were found.

Several in vitro studies have suggested that co-culturing a mixture of MSCs and chondrocytes increases matrix formation.^7,10,11^ In these mixtures, the chondrocytes could immediately start forming cartilage, and trophic effects due to the growth factors released in the system would boost this effect further.^8^ However, these in vitro studies are, by necessity, short-term studies, and it is therefore not clear how these differences develop in the longer term if they are maintained. To our knowledge, the only in vivo study used a rat model and found no difference in quality of cartilage defect repair 12 weeks after implanting scaffolds with either a 90:10 MSC:chondrocyte mixture or pure chondrocytes but did not study other time points.^12^

In Part II of our work, we aim to explore the longer term patterns over time of cartilage defect healing following implantation of mixtures of MSCs and chondrocytes at various ratios, and investigate the differences between them. The plan of the article is as follows. In the section ‘Mathematical model’, we state the model equations, boundary and initial conditions. Next, section ‘Results’ shows the results of simulations for five co-implantation ratios and their comparison with respect to matrix density levels over healing time. Results showing sensitivity to variations in co-implantation ratios are also considered here, in particular, comparisons are made with 100% stem cell (ASI) and 100% chondrocyte (ACI) implantations. Finally, section ‘Discussion’ explores the implications of the model results on co-culture cell therapy and future work. We refer the interested reader to Campbell et al.^9^ where full details of non-dimensionalisation and a sensitivity analysis of the model has been conducted, which will not be shown here.

## Mathematical model

Our mathematical model follows the same formulation as our earlier work^9^ with the initial cell implantation profile changed to accommodate a varying ratio of stem cells and chondrocytes. We only state the dimensionless equations, and boundary and initial conditions here. For more information on the formulation and non-dimensionalisation of these equations and assumptions made, the reader is referred to Campbell et al.^9^ and Lutianov et al.^5^

**Figure 1.**
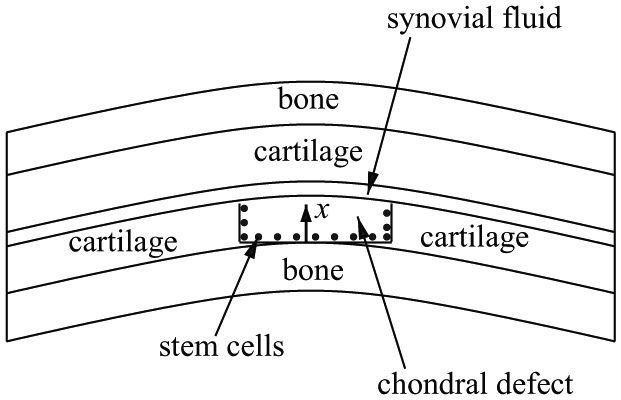
Schematic cross-section of a chondral defect. The thickness along the defect is labelled x.

We consider a cartilage defect with a small depth to diameter ratio (see Figure 1) which enables us to simplify to a one-dimensional problem where cell growth is modelled along the defect depth x only, with x=0 at the base of the defect. The variables in our model are as follows: the stem cell density $C_{S}$, the chondrocyte density $C_{C}$, the matrix density m, the nutrient concentration n, the FGF-1 concentration g and the BMP-2 concentration b. Cell density is measured in number of cells per unit volume, matrix density and growth factor concentration are measured as mass per unit volume and nutrient concentration is measured in number of moles per unit volume.

Following the non-dimensionalisation given in Campbell et al.^9^ and provided in Appendix 1, the dimensionless equations (overbars omitted), boundary and initial conditions for the evolution of the cell and matrix densities and nutrient concentration in time, t, and space, x are given by

(1a) $$\begin{array}{c} \frac{\partial C_{S}}{\partial t}=\frac{\partial }{\partial x}\left(D_{S}\left(m\right)\frac{\partial C_{S}}{\partial x}\right)\\ +p_{1}\left(m,\frac{C_{S}}{C_{S,max}\left(m\right)}\right)\frac{n}{n+n_{0}}C_{S}H\left(n-n_{1}\right)\\ -p_{2}C_{S}H\left(C_{S}-C_{S_{0}}\left(b\right)\right)-p_{3}C_{S}H\left(n_{1}-n\right) \end{array}$$

(1b) $$\begin{array}{c} \frac{\partial C_{C}}{\partial t}=\frac{\partial }{\partial x}\left(D_{C}\left(m\right)\frac{\partial C_{C}}{\partial x}\right)\\ +p_{4}\left(m,g,\frac{C_{C}}{C_{C,max}\left(m\right)}\right)\frac{n}{n+n_{0}}C_{C}H\left(n-n_{1}\right)\\ +p_{2}C_{S}H\left(C_{S}-C_{S_{0}}\left(b\right)\right)-p_{5}C_{C}H\left(n_{1}-n\right) \end{array}$$

(1c) $$\frac{\partial n}{\partial t}=D_{n}\frac{\partial^{2}n}{\partial x^{2}}-\frac{n}{n+n_{0}}\left(p_{6}C_{S}+p_{7}C_{C}\right)$$

(1d) $$\frac{\partial m}{\partial t}=D_{m}\frac{\partial^{2}m}{\partial x^{2}}+p_{8}\left(m,g\right)\frac{n}{n+n_{0}}C_{C}$$

(1e) $$\frac{\partial g}{\partial t}=D_{g}\frac{\partial^{2}g}{\partial x^{2}}+p_{9}C_{S}-p_{11}g$$

(1f) $$\frac{\partial b}{\partial t}=D_{b}\frac{\partial^{2}b}{\partial x^{2}}+p_{12}C_{C}-p_{13}b$$

where

(2) $$\begin{array}{l} p_{1}\left(m,\frac{C_{S}}{C_{S,max}\left(m\right)}\right)=A\left(m\right)\left(1-\frac{C_{S}}{C_{S,max}\left(m\right)}\right)\\ A\left(m\right)=p_{1_{0}}\frac{m}{m^{2}+m_{2}^{2}}\\ p_{4}\left(m,g,\frac{C_{C}}{C_{C,max}\left(m\right)}\right)=B\left(m\right)\left(1-\frac{C_{C}}{C_{C,max}\left(m\right)}\right)\\ B\left(m\right)=p_{4_{0}}\frac{m}{m^{2}+m_{2}^{2}}+p_{4_{00}}\frac{g}{g+1}\\ C_{S,max}\left(m\right)=C_{S,max_{0}}\left(1-m\right)\\ C_{C,max}\left(m\right)=C_{C,max_{0}}\left(1-m\right)\\ C_{S,max_{0}}+C_{C,max_{0}}=1\\ p_{8}\left(m,g\right)=\left(1-p_{8_{1}}m\right)\left(1+p_{8_{00}}\frac{g}{g+1}\right)\\ D_{S}\left(m\right)=D_{S_{0}}\frac{m}{m^{2}+m_{1}^{2}},\;D_{C}\left(m\right)=D_{C_{0}}\frac{m}{m^{2}+m_{1}^{2}}\\ C_{S_{0}}\left(b\right)=\left(C_{S_{0,max}}-C_{S_{0,min}}\right)e^{-\alpha b}+C_{S_{0,min}} \end{array}$$

The estimated values of the parameters in dimensional form and the dimensionless parameters are provided in Appendix 1 (Tables 1 and 2) and Campbell et al.^9^

The non-dimensional boundary and initial conditions are

(3a) $$\begin{array}{l} -D_{S}\left(m\right)\frac{\partial C_{S}}{\partial x}=-D_{C}\left(m\right)\frac{\partial C_{C}}{\partial x}=-D_{n}\frac{\partial n}{\partial x}\\ =-D_{m}\frac{\partial m}{\partial x}=-D_{g}\frac{\partial g}{\partial x}=-D_{b}\frac{\partial b}{\partial x}=0,\;\;\left(\mathrm{at}\;x=0\right) \end{array}$$

(3b) $$\begin{array}{l} -D_{S}\left(m\right)\frac{\partial C_{S}}{\partial x}=-D_{C}\left(m\right)\frac{\partial C_{C}}{\partial x}=-D_{m}\frac{\partial m}{\partial x}=0\\ n=1,\;\;-D_{g}\frac{\partial g}{\partial x}=\gamma g,\;\;-D_{b}\frac{\partial b}{\partial x}=\chi b,\;\;\left(\mathrm{at}\;x=1\right)\\ C_{S}=\left(1-p_{c}\right)C_{S}^{\left(0\right)}h\left(x\right),\;\;C_{C}=p_{c}C_{C}^{\left(0\right)}h\left(x\right) \end{array}$$

(3c) $$n=1,\;\;m=m_{3},\;\;g=g_{init},\;\;b=b_{init},\;\;\left(\mathrm{at}\;t=0\right)$$

with γ and χ representing the flux of growth factors leaving the top of the defect.

The new initial conditions representing the different co-culture ratios of stem cells and chondrocytes are highlighted in bold in equation (3). Here, $C_{S}^{\left(0\right)}$ and $C_{C}^{\left(0\right)}$ are the initial stem cell and chondrocyte densities, h(x) is the initial profile and $p_{c}$ ($0\;\;\;\;\;\;p_{c}\;\;\;\;\;1$) represents the proportion of chondrocytes implanted in the defect (e.g., a 35% chondrocyte proportion means $p_{c}=0.35$, a mixture consisting of 65% stem cells and 35% chondrocytes at *t* = 0).

We used a second-order accurate finite difference scheme to discretise the spatial derivatives in x over 100 grid points in equations (1) to (3), keeping the time derivative t continuous. The resulting ordinary differential equations were solved in MATLAB (Release 2013a, The MathWorks, Inc., Natick, MA, USA) using the stiff ODE solver *ode15s*. The dimensionless parameter values used in our simulations are given in Appendix Table 2.

The initial stem cell and chondrocyte density spatial profile is $C_{S}(x,0)=C_{S}^{\left(0\right)}(1-p_{c})[1-\tanh(A(x-x_{0}))]/2$ and $C_{C}(x,0)=C_{C}^{\left(0\right)}p_{c}[1-\tanh(A(x-x_{0}))]/2$, with $A{=10}^{4}$ and $x_{0}=0.1$. Dimensionally, this is equivalent to a combined chondrocyte and stem cell density of $2.5\times 10^{5}\;{\mathrm{cells}/\mathrm{mm}}^{3}$, restricted to an area of thickness 200 µmm200μmm near x=0, and zero elsewhere. We also assumed a small density of matrix $\left(m_{3}{=10}^{-4}\right)$, FGF-1 $\left(g=g_{init}\right)$ and BMP-2 $\left(b=b_{init}\right)$ uniformly distributed across the defect.

The general evolution characteristics of the cell and matrix densities, nutrient and growth factor concentrations using this model are described in Part I of this work Campbell et al.^9^ and in Lutianov et al.^5^ and hence are not repeated in detail here. The main focus of our simulations is to vary the initial stem cell and chondrocyte implantation densities through the parameter $p_{c}$, keeping the other parameters fixed.

We simulate cartilage repair following implantation of five mixtures, namely, $p_{c}=0.1$ (90% stem cells and 10% chondrocytes, hereafter referred to as 90:10), $p_{c}=0.3$ (70% stem cells and 30% chondrocytes, hereafter referred to as 70:30), $p_{c}=0.5$ (50% stem cells and 50% chondrocytes, hereafter referred to as 50:50), $p_{c}=0.7$ (30% stem cells and 70% chondrocytes, hereafter referred to as 30:70) and $p_{c}=0.9$ (10% stem cells and 90% chondrocytes, hereafter referred to as 10:90).

## Results

### Co-implantation of 90% stem cells and 10% chondrocytes

**Figure 2.**
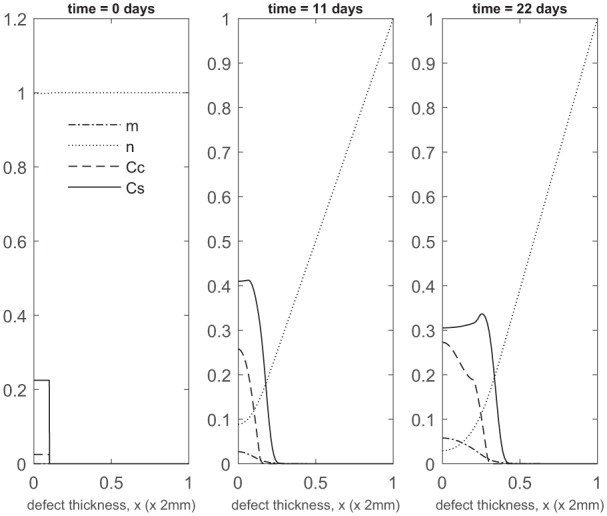
Evolution of cell and matrix densities, and nutrient concentration at times *t* = 0, 11 and 22 days following co-implantation of 90% stem cells and 10% chondrocytes.

We first show the simulations corresponding to $p_{c}=0.1$ (90% stem cells and 10% chondrocytes; 90:10). Panels 2 and 3 in Figure 2 show the evolution at *t* = 11 and 22 days, respectively. Matrix production near x=0 is seen after only a few days, mainly due to a rapid increase in chondrocyte density (almost 10 times the initial number within 11 days; see Panel 2 in Figure 2). This early matrix production is of comparable magnitude to that produced for $p_{c}=1.0$ (implantation of 100% chondrocytes; see Panel 2 in Figure 2 of Lutianov et al.^5^), but using a far smaller number of chondrocytes (see Panel 2 in Figure 2 of Lutianov et al.^5^ and Figure 19), and occurs much earlier than for $p_{c}=0$ (implantation of 100% stem cells), which requires 2 months to achieve similar matrix levels (Figure 5 in Campbell et al.^9^ and also see Figure 19).

**Figure 3.**
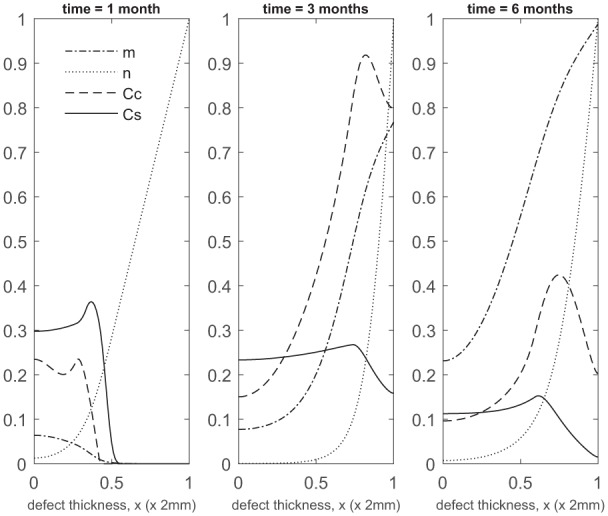
Evolution of cell and matrix densities, and nutrient concentration at times *t* = 1, 3 and 6 months following co-implantation of 90% stem cells and 10% chondrocytes.

Over the course of the first few months, chondrocyte density is generally larger in the co-implantation case compared to the 100% stem cell and 100% chondrocyte implantation cases (compare Figure 3 with Figure 5 in Campbell et al.^9^ and Figure 3 in Lutianov et al.^5^). This larger chondrocyte density comes not only with increased matrix production but also with increased uptake of nutrients. The latter results in a drop of chondrocyte density towards the bottom of the defect once the nutrient concentration falls below the minimum threshold level $n_{1}{=10}^{-1}$), increasing chondrocyte death and slowing down chondrocyte proliferation. The net result is a slowing down of matrix production at the bottom of the defect. On the other hand, chondrocyte density continues to grow at the top of the defect due to the local abundance of nutrients there, resulting in a continued increase in matrix density near the top of the defect (see Panels 2 and 3 in Figure 3).

**Figure 4.**
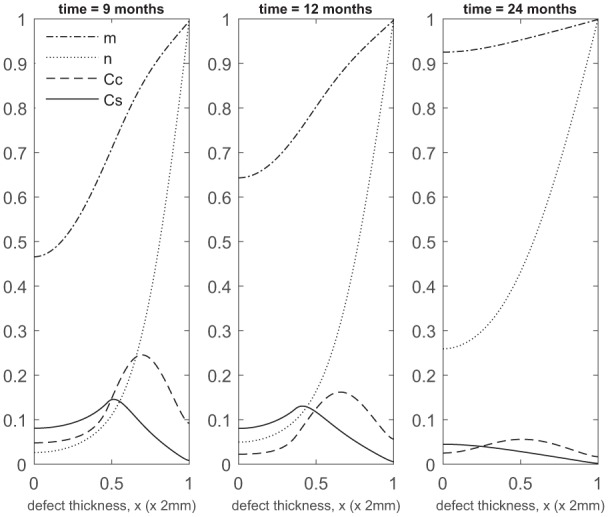
Evolution of cell and matrix densities, and nutrient concentration at times *t* = 9, 12 and 24 months following co-implantation of 90% stem cells and 10% chondrocytes.

At later times (Figure 4), matrix deposition slows down and the defect fills up in 18 to 24 months. This timescale is similar to the two single-cell-type implantation cases (Figure 4 in Lutianov et al.^5^ and Figure 6 in Campbell et al.^9^).

### Co-implantation of 70% stem cells and 30% chondrocytes

**Figure 5.**
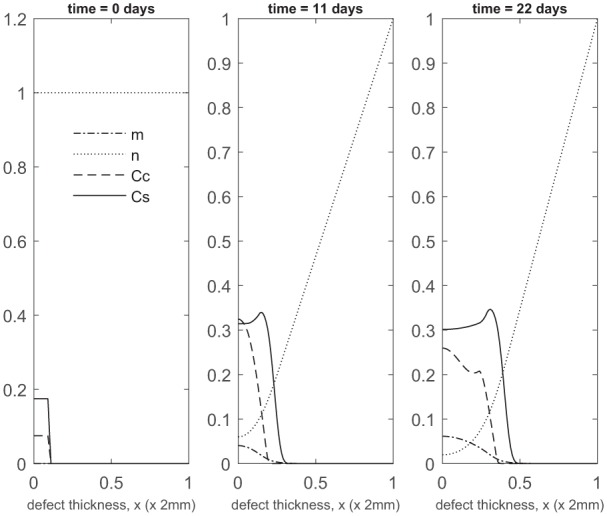
Evolution of cell and matrix densities, and nutrient concentration at times *t* = 0, 11 and 22 days following co-implantation of 70% stem cells and 30% chondrocytes.

**Figure 6.**
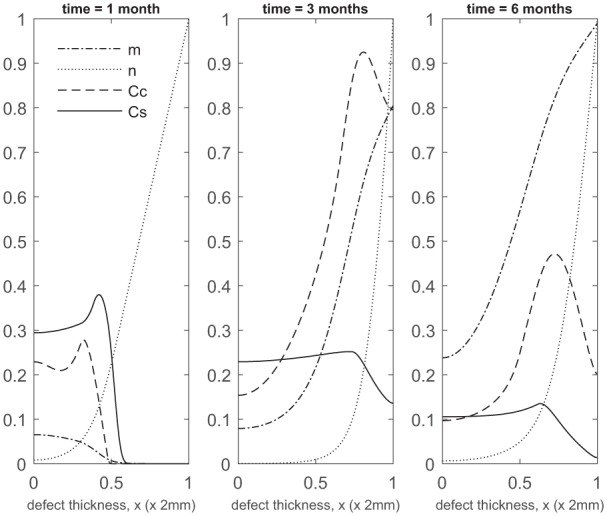
Evolution of cell and matrix densities, and nutrient concentration at times *t* = 1, 3 and 6 months following co-implantation of 70% stem cells and 30% chondrocytes.

**Figure 7.**
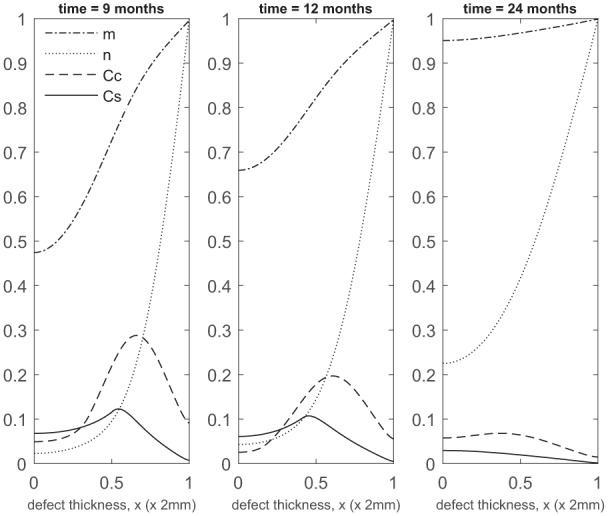
Evolution of cell and matrix densities, and nutrient concentration at times *t* = 9, 12 and 24 months following co-implantation of 70% stem cells and 30% chondrocytes.

Next we show simulations of $p_{c}=0.3$ corresponding to 70% stem cells and 30% chondrocytes (70:30). Figures 5–7 show the evolution of the cell and matrix densities and nutrient concentration for time ranging between 11 days and 24 months. Similar to the 90:10 case (Figures 2–4), we see enhanced matrix production at early time points with the nutrient concentration falling below the critical condition, $n_{1}{=10}^{-1}$, as early as 11 days at the bottom of the defect. This large consumption of nutrients is due to cell proliferation and MSC differentiation, which is enhanced due to FGF-1 and BMP-2.^8,9^ This decreases chondrocyte proliferation at the bottom of the defect, meaning diffusion of cells to higher concentrations of nutrients will be the main driver of defect healing. As time continues, we see that the general evolutionary characteristics of the simulations remain similar to our 90:10 case, albeit with slightly higher matrix levels due to the higher proportion of chondrocytes inserted into the defect. The defect is observed to fill up with new cartilage within 18 to 24 months, which is in line with our previous results.^9^

### Co-implantation of 50% stem cells and 50% chondrocytes

**Figure 8.**
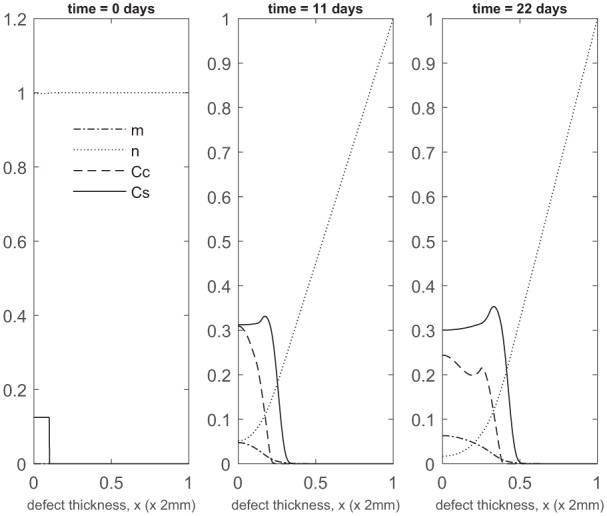
Evolution of cell and matrix densities, and nutrient concentration at times *t* = 0, 11 and 22 days following co-implantation of 50% stem cells and 50% chondrocytes.

**Figure 9.**
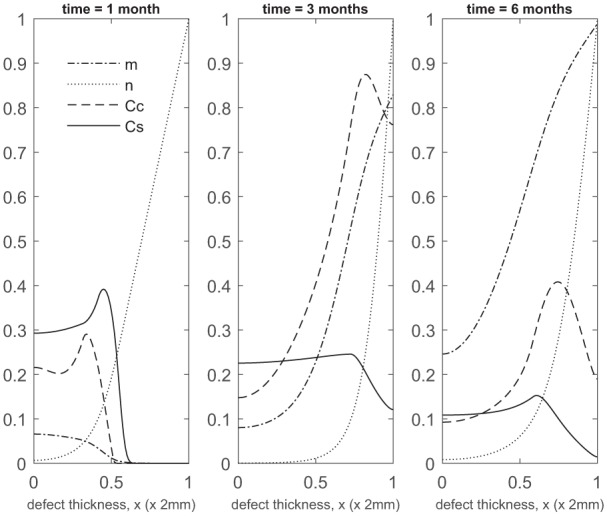
Evolution of cell and matrix densities, and nutrient concentration at times *t* = 1, 3 and 6 months following co-implantation of 50% stem cells and 50% chondrocytes.

**Figure 10.**
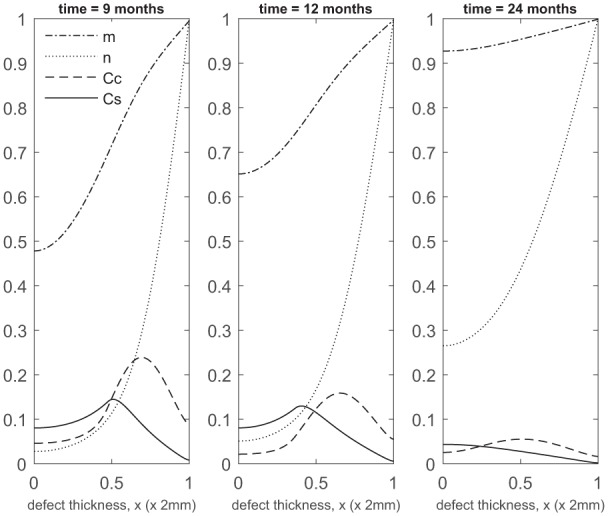
Evolution of cell and matrix densities, and nutrient concentration at times *t* = 9, 12 and 24 months following co-implantation of 50% stem cells and 50% chondrocytes.

We next show the simulations corresponding to $p_{c}=0.5$ (50% stem cells and 50% chondrocytes; 50:50). Figures 8–10 show the evolution of the cell and matrix densities and nutrient concentration for this case at early and late time points. The evolution characteristics are identical to the 90:10 and 70:30 cases, except that the overall matrix density is slightly higher, particularly at earlier times (compare Panel 2 Figure 8 and Figure 2). This is a consequence of the larger proportion of implanted chondrocytes and the subsequent increase in chondrocyte density due to a combination of growth factor enhanced proliferation and stem cell differentiation. However, at later time points, the increased nutrition demand from the larger overall cell density causes the nutrient concentration close to the bottom of the defect to fall below the minimum threshold level $n_{1}{=10}^{-1}$, in turn slowing down cell proliferation and matrix production rates. Thus, the matrix density at later times is very similar to the 90:10 and 70:30 cases (compare Figure 9 with Figures 3 and 6).

### Co-implantation of 30% stem cells and 70% chondrocytes

**Figure 11.**
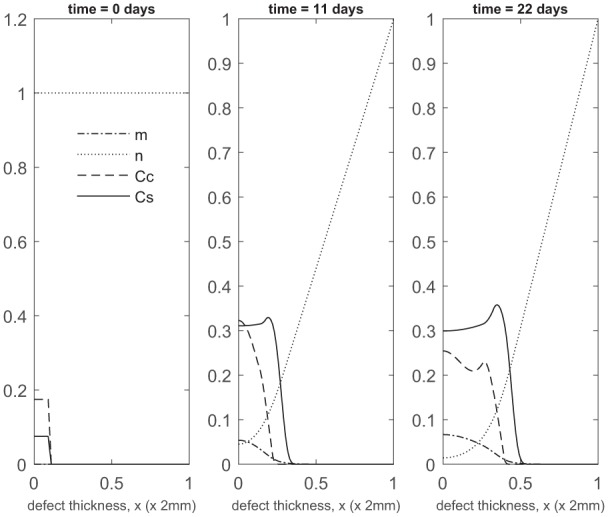
Evolution of cell and matrix densities, and nutrient concentration at times *t* = 0, 11 and 22 days following co-implantation of 30% stem cells and 70% chondrocytes.

**Figure 12.**
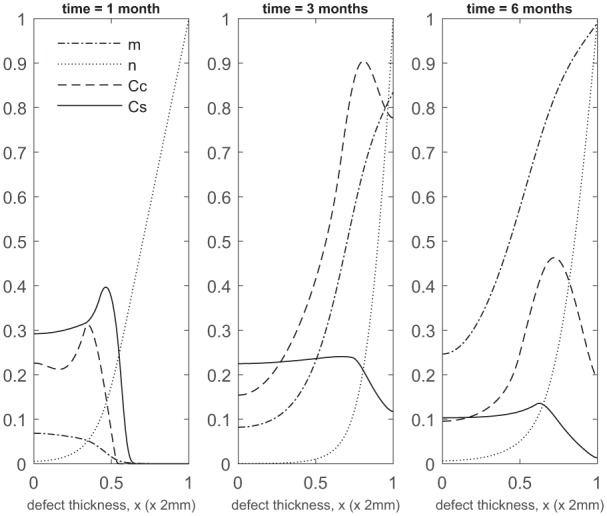
Evolution of cell and matrix densities, and nutrient concentration at times *t* = 1, 3 and 6 months following co-implantation of 30% stem cells and 70% chondrocytes.

**Figure 13.**
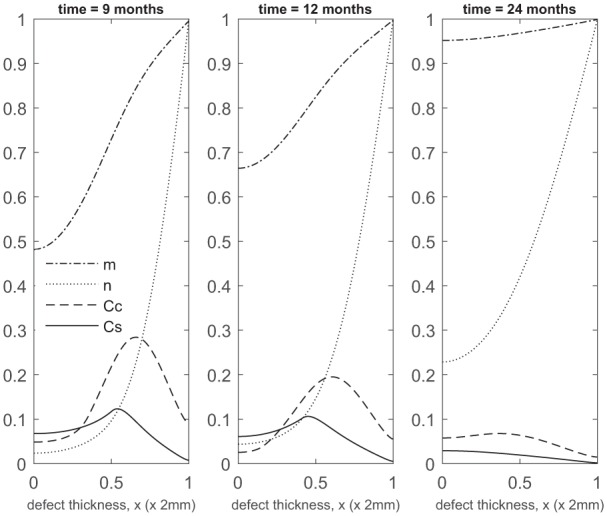
Evolution of cell and matrix densities, and nutrient concentration at times *t* = 9, 12 and 24 months following co-implantation of 30% stem cells and 70% chondrocytes.

Figures 11–13 show cell and matrix densities, and nutrient concentration for $p_{c}=0.7$ simulations corresponding to 30% stem cells and 70% chondrocytes (30:70). Here we observe high levels of matrix at early times. As with the other cases, nutrients are a limiting factor on healing, falling below the critical concentration and switching off cell proliferation by 11 days. MSCs appear to begin diffusing towards the top of the defect sooner in this case when compared with the 90:10 case (Figure 2), for instance, likely to be due to higher matrix density allowing for cell motility. Once cell diffusion to the top of the defect has begun, we observe similar trends to the previous cases (Figures 3, 6 and 9). By 9 months (Figure 13), matrix densities are similar to those of our previous cases (Figures 4, 7 and 10), indicating that the differences we see at early times are not maintained as time continues. This could be due to limited nutrient concentration, which is consistently low during the evolution.

### Co-implantation of 10% stem cells and 90% chondrocytes

**Figure 14.**
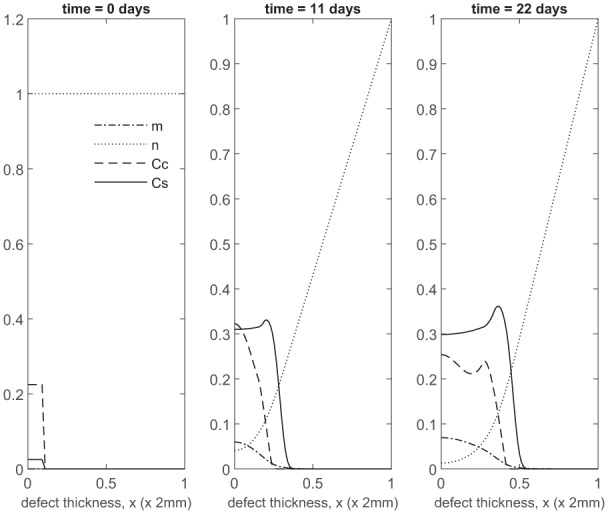
Evolution of cell and matrix densities, and nutrient concentration at times *t* = 0, 11 and 22 days following co-implantation of 10% stem cells and 90% chondrocytes.

**Figure 15.**
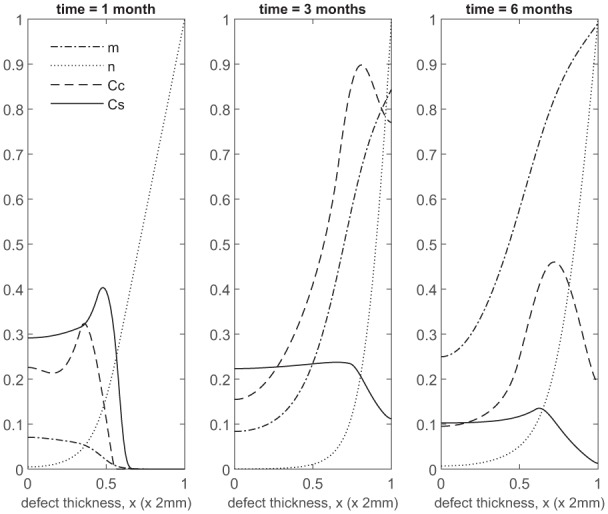
Evolution of cell and matrix densities, and nutrient concentration at times *t* = 1, 3 and 6 months following co-implantation of 10% stem cells and 90% chondrocytes.

**Figure 16.**
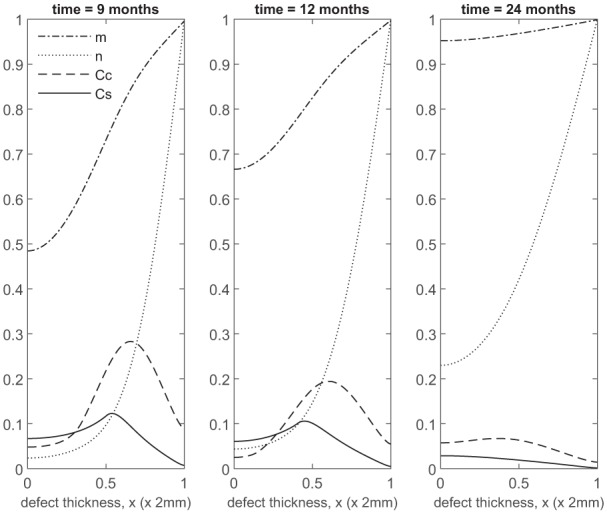
Evolution of cell and matrix densities, and nutrient concentration at times *t* = 9, 12 and 24 months following co-implantation of 10% stem cells and 90% chondrocytes.

We finally show the results for a 90% chondrocyte and 10% MSC mixture corresponding to $p_{c}=0.9$ (10:90) (Figures 14–16). Here we have the highest proportion of chondrocytes inserted into the defect and as such have the highest matrix levels at early times. This is likely due to increased matrix formation primarily occurring at early times during our simulations, when nutrients are more readily available in the defect. This means a higher implanted chondrocyte density, as demonstrated here, could be desirable to increase matrix levels. Despite this, as with our previous co-implantation cases, increased matrix deposition appears to slow at later times, with nutrient concentration and cell diffusion being the main regulatory factors of healing.

Next, we make a comparison between the five co-implantation cases with ACI and ASI to identify both spatial and temporal differences in matrix and cell densities.

### Comparison of matrix density of co-implantation, ACI and ASI at early times

**Figure 17.**
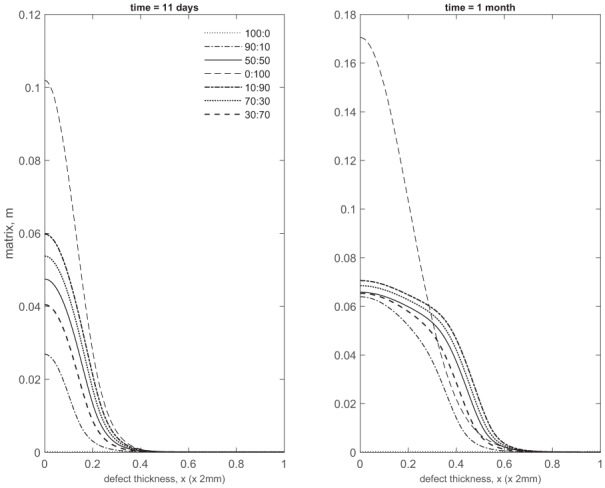
Comparison of matrix density profiles for all cases at times *t* = 11 days and 1 month.

**Figure 18.**
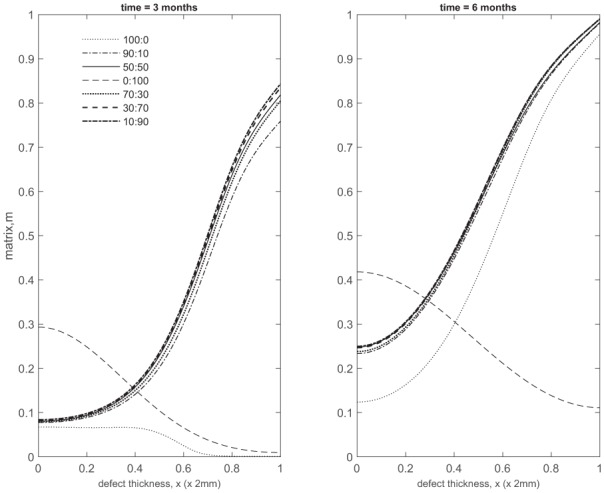
Comparison of matrix density profiles for all cases at times *t* = 3 and 6 months.

Figures 17 and 18 compare matrix densities at early times for five co-implantation cases with ACI and ASI.

Up to 1 month, the 100% chondrocyte case (0:100) has the largest amount of matrix (Figure 17). Although at 11 days the chondrocyte density in the 90:10 case is close to that of other co-implantation cases containing higher chondrocyte densities, and even higher than in the 0:100 case (compare Figures 2, 5, 8, 11 and 14, and Figure 2 in Lutianov et al.^5^), the additional nutrient demands of the stem cells bring the nutrient concentration below the minimum threshold value, resulting in matrix densities much lower than the 0:100 case (Figure 17). In the 100:0 case, the stem cells have not yet differentiated into chondrocytes at these early time points, and hence no matrix at all is produced (Figure 17).

The 10:90 case has the highest level of matrix at 3 months (Panel 1 in Figure 18), consistent with the observations in Figures 14, 15 and 16. The five co-implantation cases produce more matrix than the 0:100 case, despite the 0:100 case having the largest matrix density at earlier times and the highest implantation of chondrocytes. The 100:0 implantation case, relevant to ASI, still has the lowest matrix levels, indicating that the implantation of MSCs alone delays healing initially (Panel 1 in Figure 18).’ Can be changed to ‘case’ instead of ‘implantation case’ if required.

These findings highlight the importance of early matrix deposition, as it is clear at late times that the differences we observe in matrix levels between our co-implantation cases are more moderate (Figures 4, 7 10, 13 and 16, Panel 2 in Figure 18). At late times, our simulations are more likely to be constrained by low nutrient concentrations, therefore slowing the rate of healing down. At early times, more nutrients are available within the defect, primarily at the top, where formation of cartilage is most notable in our ASI and co-implantation cases. We find our ACI case forms matrix primarily at the bottom of the defect as nutrient levels never become very low here, unlike for our other cases, meaning cells are not forced to diffuse to areas of higher nutrient concentration to continue proliferating (Figure 18). Chondrocytes also have a lower cell motility rate in comparison to MSCs, meaning diffusion to the top of the defect will be slower.

### Comparing mean cell and matrix densities versus time for co-implantation, ACI and ASI

Here we compare the mean matrix, chondrocyte and MSC densities over a period of 24 months for four cases: 0:100 (ACI), 100:0 (ASI) and two co-implantation strategies, 90:10 and 10:90. We choose to focus on 90:10 and 10:90 as they represent our two most extreme co-implantation cases, with all other results, that is, 70:30, 50:50 and 30:70, lying within the bounds of these two sets of results (see Figures 17 and 18). The two single-cell implantation cases are investigated in Lutianov et al.^5^ and Part I of our work Campbell et al.,^9^ and the interested reader is referred to these studies.

**Figure 19.**
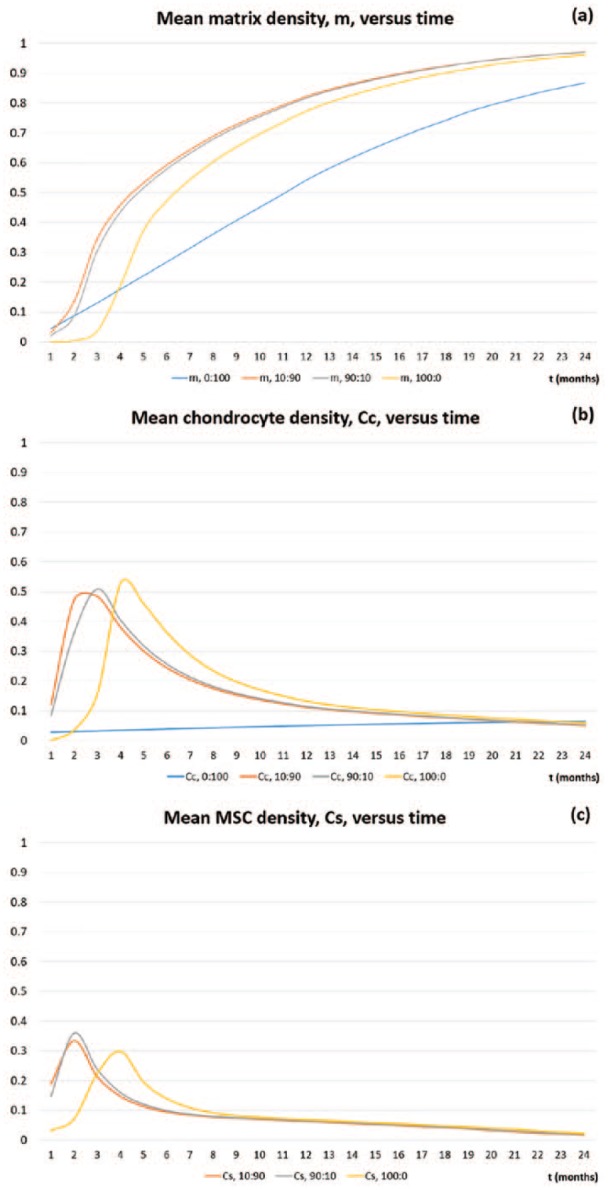
Mean densities of (a) matrix m, (b) chondrocytes $C_{C}$ and (c) MSCs $C_{S}$, as a function of the time, in months, from 1 to 24 months for 0:100 (ACI, blue), 10:90 (orange), 90:10 (grey) and 100:0 (ASI, yellow).

In Figure 19(a), at 1 month, the mean matrix density produced is largest for the 0:100 case (blue). This is not only because this case has the largest concentration of chondrocytes directly producing matrix from the beginning but also because only chondrocytes are seeded in the defect. The co-implantation cases also have a population of stem cells competing for nutrients, thus reducing the average matrix production by the chondrocytes. At 2 months, the 100:0 (grey) case has produced barely any matrix due to MSCs having to first differentiate into chondrocytes before matrix deposition can begin. Also at this time, our co-implantation cases (90:10 grey, 10:90 orange) have already surpassed the matrix levels of 0:100 despite containing less implanted chondrocytes. This is due to growth factors being released by the cell-to-cell interaction of the MSCs and chondrocytes^9^ and the balance of the effects of cell proliferation and nutrient levels. In our model, MSCs have a high demand for nutrients to support their high proliferation rate and their differentiation into chondrocytes. In the 90:10 case, the large concentration of MSCs therefore consumes a large amount of nutrients, leaving less for the chondrocytes to produce matrix. On the other hand, in the 10:90 case, the MSC density is lower, and therefore these cells consume less nutrients, leaving more nutrients for the chondrocytes to proliferate and deposit matrix. This difference is mainly observable at early times.

At 3 months, the mean matrix density for the 90:10 (grey) case is 136% higher than the 0:100 case (blue), and has an even higher percentage difference when compared with the 100:0 case (yellow). This marked increase in matrix density is due to the effects of the growth factors.^9^ We see a higher percentage difference when compared to 100:0 due to lower mean chondrocyte density at this time compared to 90:10 and 0:100 (see Figure 19(b) for the mean chondrocyte density comparison between the co-implantation cases and ACI and ASI). Beyond 3 months, this increase in mean matrix levels is sustained for the co-implantation cases with an 80% increase at 12 months when compared with 0:100 for our 90:10 case. The percentage difference is smaller when compared with the 100:0 case, with a 5.5% increase at 12 months. Past 1 year, we see that the co-implantation cases maintain the highest mean matrix levels, which is an accumulation of the differences in matrix levels at early times.

In Figure 19(b), we compare the mean chondrocyte densities for the 10:90 (orange), 90:10 (grey) and 100:0 (yellow) cases. We do not show the evolution of the mean cell density of the 0:100 case since it is more localised to the bottom of the defect, and therefore not a good comparison for mean cell levels. We see at 1 month that the 10:90 case (orange) has the highest level of chondrocytes, but despite this matrix deposition is slow initially due to nutrient levels falling below the critical condition, $n_{1}{=10}^{-1}$ (Figure 9). This effect is also observable in the 90:10 case (orange). At 3 months, chondrocyte levels have increased dramatically in our co-implantation cases, indicating MSC differentiation has been initiated, thus leading to these cases having the highest matrix density (Figure 19(a)).

In Figure 19(c), we compare the mean MSC densities for the 10:90 (grey) and 90:10 (orange) co-implantation and 100:0 (yellow) cases. The 0:100 contains no MSCs. At 1 month, the 90:10 case has the highest density of MSCs, despite the 100:0 case having the highest implantation of MSCs. In the 10:90 and 90:10 cases, cell-to-cell interaction releases growth factors almost immediately, meaning chondrocyte proliferation and MSC differentiation are enhanced.^8,9^ This is likely to be the cause of the marked increase in MSC levels in the defect at this time.

## Discussion

This study aimed to develop a mathematical model to explore the longer term patterns over time of cartilage defect healing following implantation of mixtures of MSCs and chondrocytes at various ratios, and investigate the differences between them. First, our simulations suggest that co-implanting MSCs and chondrocytes will increase matrix deposition within the first half year of healing when compared with 100% MSC (ASI) or 100% chondrocyte (ACI) implantation therapies, indicating a chondral defect could fill with new cartilage at earlier times when a co-culture procedure is the chosen treatment. Although 10:90 appears to have the highest matrix density at early times, clinically a co-implantation ratio that uses less chondrocytes is desirable if the aim would be to develop a single-stage ACI procedure.^11^ Opting for the lower proportion of chondrocytes in these co-implantation therapies could mean sufficient chondrocytes can be isolated from the cartilage harvest obtained during arthroscopy for a successful co-implantation procedure.^13^ This alleviates the need for expansion of cells in vitro if the fresh chondrocytes are combined with allogeneic stem cells, allowing cells to be harvested and inserted into the defect region during one procedure.^14^ Alternatively, the fresh chondrocytes can be mixed with fresh bone marrow, which despite the lower total cell number has been suggested to be clinically effective.^15^

Our model enabled us to compare matrix densities following co-implantation of MSCs and chondrocytes at various ratios, not only visualising the cartilage matrix density distribution at any time point but also investigating how the concentrations of MSCs, chondrocytes and nutrients change within the defect in response to different co-implantation ratios. The five ratios we focused on were 90% MSCs plus 10% chondrocytes, 70% MSCs plus 30% chondrocytes, 50% MSCs plus 50% chondrocytes, 30% MSCs plus 70% chondrocytes and 10% MSCs plus 90% chondrocytes, with 90:10 and 50:50 having been or are being investigated clinically.^14,16^ We compared these to ACI (100% chondrocytes) and ASI (100% MSCs). When comparing co-implantation scenarios with the ACI and ASI results from our previous work,^5,9^ it is clear that a mixture of MSCs and chondrocytes delivers the desired effect of increased matrix deposition, as hypothesised in the literature^17^ and in previous experiments.^8^ This effect is especially marked during the first few months following cell implantation, but from around sixth month onwards, the differences, especially with ASI, become small. As time progresses, the 0:100 case continues to produce matrix at a steady rate, but the 100:0 and co-implantation cases soon surpass these levels. Figure 19(a) shows how total matrix levels of 100:0 (ASI), 90:10, 10:90 and 0:100 (ACI) simulations compare at over a period of 2 years. At early time, there is a monotonic increase in the total matrix density with the 0:100 case having the highest density, 100:0 having produced almost no matrix at all and the co-implantation cases having almost similar intermediate levels of matrix. This indicates that at early time, chondrocyte proliferation balanced with adequate nutrient availability is the main identifiable mechanism responsible for the formation of new cartilage in our model. As time progresses, 0:100 continues to produce matrix at a steady rate, but 100:0 and co-implantation cases soon surpass these levels. Beyond 6 months, there is a non-monotonic increase in the total matrix density with a peak in matrix levels in the co-implantation cases, and the 100:0 case still producing the lowest level of matrix. Although we cannot say with any certainty that the maximum matrix density is obtained precisely for the 10:90 or 90:10 case, there is a definite optimal ratio of stem cells and chondrocytes that can produce maximum matrix at intermediate times. This indicates that at these times, cell differentiation and diffusion are the important mechanisms driving new cartilage formation. From 6 months onwards, we found little difference in the distribution of cell types and cartilage matrix between the five co-implantation cases and implanting only stem cells. This suggests that implanting a cell population that includes stem cells will lead to a stable solution path, regardless of the exact proportion of stem cells. Although co-implantation of chondrocytes and stem cells led to more matrix deposition at earlier time points, this difference was not maintained, and by 12 months, the difference in matrix production between the five cases was very small. Similar small differences have been found between 1-year biopsies obtained in human trials of co-implanted cells, stem cells or chondrocytes.^2,14^ Nevertheless, the larger matrix deposition at earlier time may give advantages with respect to the rehabilitation, which could be faster if matrix is formed earlier. This alone could be an important clinical advance in the treatment of articular cartilage damage.

A mixture of stem cells and chondrocytes produces more consistent levels of matrix due to the balance of nutrients used between the two cell types and the release of important growth factors that influence chondrocyte proliferation and stem cell differentiation. In our model, this effect is partly due to the cell–cell interactions between MSCs and chondrocytes, releasing growth factors such as FGF-1 and BMP-2 that cause an increase in matrix deposition from increased chondrocyte proliferation and enhanced chondrogenesis.^8^ In addition, the increase of matrix deposition and chondrocyte density at early times for our co-implantation cases is in part due to the lower proliferation rate of the chondrocytes, allowing more nutrients to be available in the defect for MSC proliferation and differentiation.

An important assumption in our model concerns the role of chondrogenesis, the differentiation of stem cells into chondrocytes. Our results suggest that stem cell differentiation played an important part in increasing the number of chondrocytes, and eventually the matrix, due to large quantities of chondrocytes, comparable to our 50:50 case, being present in the defect when 90% MSCs are implanted. Most in vitro co-culture studies suggest that the more important contribution from the stem cells is their positive effect on chondrocyte proliferation whereas their differentiation into chondrocytes is less important.^7^

Other mixtures of MSCs and chondrocytes could be investigated to find an ‘optimal’ MSC/chondrocyte ratio, where nutrient constraints are minimised and matrix deposition maximised. Our criterion for suggesting an optimal co-implantation ratio is based on mean matrix densities. However, other criteria could also be used to determine an optimal ratio. Some justifications of our current criterion are clinical data comparing magnetic resonance imaging (MRI) and clinical outcome that suggests the signal intensity on MRI correlates with better clinical outcome of ACI.^18^ The signal intensity is a measure of mean matrix density, and thus our chosen measure will give a clinically relevant comparison. However, other parameters such as the required time for cartilage matrix to fill the defect and the required time to achieve a threshold density at the surface might also be appropriate. The spatial distribution of matrix might also be relevant, as seen in Figures 17 and 18. However, our results suggest that this may be difficult to translate in a criterion. The comparison between MRI and clinical outcome suggests that the articular surface of the repair tissue may be most important,^18^ which would suggest that the repairs including stem cells, which form denser matrix at the defect surface, might be better. However, the distribution of matrix density is less homogeneous for these cases, and poor matrix homogeneity is associated with poorer clinical outcome.^18^ The limitations of our model dictate that all simulations are subject to nutrient concentration constraints, typically meaning an optimal split of MSCs and chondrocytes is not at all obvious; this would require further investigation. This effect of nutrient concentration impacting the overall healing process has been hypothesised in our previous model as well as similar work,^19^ with this co-implantation model now corroborating this hypothesis further. Availability of cell types, overall cost and efficacy of the procedure are factors that would also have to be considered when considering an optimal MSC–chondrocyte co-implantation ratio.

## Appendix 1

### Non-dimensionalisation and estimates of dimensional and dimensionless parameters

The estimated values of the dimensional parameters appearing in the model and the references from which they are obtained are provided in Table 1.

**Table 1.** Estimated values of dimensional parameters.

Dimensional parameters	Estimated values
Defect thickness, d	2–3 mm
Maximum stem cell migration (or diffusion) coefficient, $D_{S}$^20^	3.6 × (10^−4^–10^−3^) mm^2^/h
Maximum chondrocyte migration (or diffusion), constant, $D_{C}$^20^	3.6 × 10^−4^ mm^2^/h
Stem cell migration (or diffusion), constant, $D_{S_{0}}=2m_{1}D_{S}$	7.2 × (10^−9^–10^−8^) (mm^2^/h) (g/mm^3^) (assuming $m_{1}{=10}^{-5}\;g/{\mathrm{mm}}^{3}$)
Chondrocyte migration (or diffusion), constant, $D_{C_{0}}=2m_{1}D_{C}$	7.2 × 10^−9^ (mm^2^/h) (g/mm^3^) (assuming $m_{1}{=10}^{-5}\;g/{\mathrm{mm}}^{3}$)
Nutrient diffusion coefficient, $D_{n}$^21^	4.6 mm^2^/h
Matrix diffusion coefficient, $D_{m}$^20^	2.5 × 10^−5^ mm^2^/h
FGF-1 diffusion coefficient, $D_{g}$^22^	2 × 10^−3^ mm^2^/h
BMP-2 diffusion coefficient, $D_{b}$^22^	2 × 10^−3^ mm^2^/h
Maximum stem cell proliferation rate, $p_{1}$^22^	0.2 cell/h or 5 cells/day
Stem cell proliferation constant, $p_{1_{0}}=2m_{2}p_{1}$	4 × 10^−6^ g/mm^3^/h (assuming $m_{2}{=10}^{-5}\;g/{\mathrm{mm}}^{3}$)
Stem cell differentiation rate, $p_{2}$^20^	3.75 × 10^−3^/h
Stem cell death rate, $p_{3}$	3.75 × 10^−3^/h (guess)
Maximum chondrocyte proliferation rate, $p_{4}$	2 × 10^−4^/h (guess)
Chondrocyte proliferation constant, $p_{4_{0}}=2m_{2}p_{4}$	4 × 10^−9^ g/mm^3^/h
Chondrocyte death rate, $p_{5}$	3.75 × 10^−3^/h (guess)
FGF-1 production constant, $p_{9}$	10^−17^ (g/mm^3^)/((Nc/mm^3^) h) (guess)
BMP-2 production constant, $p_{12}$	10^−17^ (g/mm^3^)/((Nc/mm^3^) h) (guess)
FGF-1 degradation rate, $p_{11}$	5.8 × 10^−2^/h (based on 12 h half-life guess)
BMP-2 degradation rate, $p_{13}$	5.8 × 10^−2^/h (based on 12 h half-life guess)
Chondrocyte proliferation rate (from FGF-1), $p_{4_{00}}$	2 × 10^−4^/h (guess)
Matrix production constant, $p_{8_{0}}$^20^	3.75 × 10^−13^ (g/mm^3^)/((Nc/mm^3^) h)
Matrix degradation constant, $p_{8_{1}}$^20^	3.75 × 10^−13^ (g/mm^3^)/((Nc/mm^3^) h)
Nutrient uptake constant by stem cells, $p_{6}$^21^	1.5 × 10^−14^ Nm/(Nc h)
Nutrient uptake constant by chondrocytes, $p_{7}$^21^	1.5 × 10^−14^ Nm/(Nc h)
FGF-1 matrix deposition rate, $p_{8_{00}}$	0–1 (guess)
Maximum total cell density, $C_{total,max_{0}}$	10^6^ Nc/mm^3^ (assuming 10 µm cell diameter)
Maximum stem cell density, $C_{S,max_{0}}$	0–10^6^ Nc/mm^3^
Maximum chondrocyte density, $C_{C,max_{0}}$	0–10^6^ Nc/mm^3^
Maximum matrix density, $m_{max}$^22^	10^−4^ g/mm^3^
Initial stem cell density, $C_{S}^{\left(0\right)}$	2.5 × 10^5^ Nc/mm^3^ (based on 10^6^ cells in 20 mm × 20 mm × 10 µm volume)
Initial cartilage cell density, $C_{C}^{\left(0\right)}$	10^2^ Nc/mm^3^ (10^−2^% of total cell density)
Threshold stem cell density, $C_{S_{0max}}$	$C_{total,max_{0}}/2\;\mathrm{Nc}/{\mathrm{mm}}^{3}$ (guess)
Threshold stem cell density, $C_{S_{0min}}$	90% of $C_{S_{0max}}$ (guess)
Matrix density, $m_{1}$	10^−5^ g/mm^3^ (assumed $m_{max}/10$)
Matrix density, $m_{2}$	10^−5^ g/mm^3^ (assumed $m_{max}/10$)
Initial matrix density, $m_{3}$	10^–8^ g/mm^3^ (assumed $m_{max}{/10}^{4}$)
Initial nutrient concentration, $N_{0}$^21^	$(2.85-9.5)\times 10^{-11}\mathrm{Nm}/{\mathrm{mm}}^{3}$
Initial FGF-1 concentration, $g_{init}$^22^	10^−12^ g/mm^3^
Initial BMP-2 concentration, $b_{init}$^22^	10^−12^ g/mm^3^
Threshold nutrient concentration, $n_{0}$^21^	$2.3\times 10^{-11}\mathrm{Nm}/{\mathrm{mm}}^{3}$
Critical nutrient concentration, $n_{1}$	$9.5\times 10^{-12}\mathrm{Nm}/{\mathrm{mm}}^{3}$ (assumed $N_{0}/10$)
Threshold stem cell density reduction factor, α	10^10^/(g/mm^3^) (guess)
FGF-1 reference concentration, $g_{0}$^22^	10^−10^ g/mm^3^
BMP-2 reference concentration, $b_{0}$^22^	10^−10^ g/mm^3^
FGF-1 flux coefficient, γ	10^−2^ mm/h (guess)
BMP-2 flux coefficient, χ	10^−2^ mm/h (guess)

Nc represents number of cells and Nm is number of moles.

We non-dimensionalise the variables as follows

(4) $$\begin{array}{l} x=x/d,\;t=t\left(p_{8_{0}}C_{total,max_{0}}/m_{max}\right)\\ \left(C_{S},C_{C}\right)=\left(C_{S},C_{C}\right)/C_{total,max_{0}},\;m=m/m_{max}\\ n=n/N_{0},\;g=g/g_{0},\;b=b/b_{0} \end{array}$$

The characteristic quantities used to measure the spatial variable x, cell densities, matrix density and nutrient and growth factor concentrations are the defect thickness d, the reference maximum total cell density $C_{total,max0}$, the maximum matrix density $m_{max}$, the initial nutrient concentration $N_{0}$ and reference growth factor concentrations $g_{0}$ and $b_{0}$, respectively. We choose to measure time, t, based on the matrix production timescale, $m_{max}/(p_{8_{0}}C_{total,max_{0}})$. Using the parameter values in Table 1, we estimate this timescale to be approximately 11 days (a unit of time corresponds to approximately 11 days). The dimensionless parameters appearing in the model and their estimated values are provided in Table 2.

**Table 2.** Estimated values of dimensionless parameters.

Dimensionless parameters	Estimated values
Stem cell migration (or diffusion) constant, $D_{S_{0}}=D_{S_{0}}/(p_{8_{0}}C_{total,max_{0}}d^{2})$	10^−3^–10^−2^
Chondrocyte migration (or diffusion) constant, $D_{C_{0}}=D_{C_{0}}/(p_{8_{0}}C_{total,max_{0}}d^{2})$	10^−3^
Nutrient diffusion coefficient, $D_{n}=D_{n}m_{max}/(p_{8_{0}}C_{total,max_{0}}d^{2})$	(1–3) × 10^2^
Matrix diffusion coefficient, $D_{m}=D_{m}/(p_{8_{0}}C_{total,max_{0}}d^{2})$	10^−3^–10^−2^
FGF-1 diffusion coefficient, $D_{g}=D_{g}m_{max}/(p_{8_{0}}C_{total,max_{0}}d^{2})$	1.14
BMP-2 diffusion coefficient, $D_{b}=D_{b}m_{max}/(p_{8_{0}}C_{total,max_{0}}d^{2})$	1.14
Stem cell proliferation constant, $p_{1_{0}}=p_{1_{0}}/(p_{8_{0}}C_{total,max_{0}})$	12
Stem cell differentiation rate, $p_{2}=p_{2}m_{max}/(p_{8_{0}}C_{total,max_{0}})$	1
Stem cell death rate, $p_{3}=p_{3}m_{max}/(p_{8_{0}}C_{total,max_{0}})$	1
Chondrocyte proliferation constant, $p_{4_{0}}=p_{4_{0}}/(p_{8_{0}}C_{total,max_{0}})$	0.012
Chondrocyte death rate, $p_{5}=p_{5}m_{max}/(p_{8_{0}}C_{total,max_{0}})$	1
FGF-1 production constant, $p_{9}=p_{9}m_{max}/(p_{8_{0}}g_{0})$	26.67
FGF-1 degradation rate, $p_{11}=p_{11}m_{max}/(p_{8_{0}}C_{total,max_{0}})$	15.4
BMP-2 production constant, $p_{12}=p_{12}m_{max}/(p_{8_{0}}b_{0})$	26.67
BMP-2 degradation rate, $p_{13}=p_{13}m_{max}/(p_{8_{0}}C_{total,max_{0}})$	15.4
Chondrocyte proliferation rate (from FGF-1), $p_{4_{00}}=p_{4_{00}}m_{max}/(p_{8_{0}}C_{total,max_{0}})$	0.012
Matrix degradation constant, $p_{8_{1}}=p_{8_{1}}m_{max}/p_{8_{0}}$	1
Nutrient uptake constant by stem cells, $p_{6}=p_{6}m_{max}/(p_{8_{0}}N_{0})$	10^4^
Nutrient uptake constant by chondrocytes, $p_{7}=p_{7}m_{max}/(p_{8_{0}}N_{0})$	10^4^
FGF-1 matrix deposition rate, $p_{8_{00}}$	0–1
Threshold nutrient concentration, $n_{0}=n_{0}/N_{0}$	0.24–0.81
Critical nutrient concentration, $n_{1}=n_{1}/N_{0}$	0.1
Threshold stem cell density, $C_{S_{0max}}=C_{S_{0max}}/C_{total,max_{0}}$	0.35
Threshold stem cell density, $C_{S_{0min}}=C_{S_{0min}}/C_{total,max_{0}}$	0.315
Initial stem cell density, $C_{S}^{\left(0\right)}=C_{S}^{\left(0\right)}/C_{total,max_{0}}$	0.25
Initial chondrocyte density, $C_{C}^{\left(0\right)}=C_{C}^{\left(0\right)}/C_{total,max_{0}}$	10^−4^
Maximum stem cell density, $C_{S,max_{0}}=C_{S,max_{0}}/C_{total,max_{0}}$	0.6
Maximum chondrocyte density, $C_{C,max_{0}}=C_{C,max_{0}}/C_{total,max_{0}}$	0.4
Matrix density, $m_{1}=m_{1}/m_{max}$	10^−1^
Matrix density, $m_{2}=m_{2}/m_{max}$	10^−1^
Initial matrix density, $m_{3}=m_{3}/m_{max}$	10^−4^
Initial FGF-1 concentration, $g_{init}=g_{init}/g_{0}$	10^−2^
Initial BMP-2 concentration, $b_{init}=b_{init}/b_{0}$	10^−2^
FGF-1 flux coefficient, $\gamma =\gamma /(p_{8_{0}}C_{total,max_{0}}d/m_{max})$	1
BMP-2 flux coefficient, $\chi =\chi /(p_{8_{0}}C_{total,max_{0}}d/m_{max})$	1
Threshold stem cell density reduction factor, $\alpha =\alpha b_{0}$	100
